# Lotka–Volterra dynamics facilitate sustainable biocontrol of wastewater sludge bulking

**DOI:** 10.1093/ismejo/wraf235

**Published:** 2025-10-23

**Authors:** Fabienne Baltes, Antonia Weiss, Marina Ettl, Kenneth Dumack

**Affiliations:** Terrestrial Ecology, University of Cologne, Zülpicher Straße 47b, 50674 Cologne, Germany; Terrestrial Ecology, University of Cologne, Zülpicher Straße 47b, 50674 Cologne, Germany; Schöttroy 13, 46519 Alpen, Germany; Terrestrial Ecology, University of Cologne, Zülpicher Straße 47b, 50674 Cologne, Germany; Aquatic Ecosystem Analyses, Institute for Integrated Natural Sciences, Universitätsstraße 1, 56070 Koblenz, University of Koblenz, Germany

**Keywords:** sludge foaming, wastewater treatment, predator–prey, testate amoebae, Arcellinida, Microthrix

## Abstract

Biological wastewater treatment is driven by complex interactions between prokaryotes and eukaryotes. Occasionally, filamentous bacteria species, first and foremost Ca. Microthrix parvicella, increase in abundance and lead to detrimental wastewater sludge bulking or floating, causing environmental harm and financial losses. Current mitigation strategies rely heavily on nonspecific chemical interventions, which present environmental risks and lack prolonged effectiveness. Here, we utilise long-term monitoring data from four German wastewater treatment plants to explore sustainable biocontrol alternatives. Our findings reveal Lotka–Volterra dynamics between Ca. Microthrix parvicella and the protist *Arcella* spp. Visual and experimental validation demonstrate the suppression of filamentous bacterial growth by predation. We further model these interactions, predicting the biocontrol potential of *Arcella* spp*.* for both immediate and sustained efficacy in managing sludge bulking. These results highlight the potential of protists as biological control agents, providing a more sustainable and environmentally friendly alternative to chemical treatment.

## Introduction

Water is a precious and finite resource, and the utilisation of microorganisms in wastewater treatment and reclamation holds immense significance. To recycle wastewater, societies depend on wastewater treatment plants (WWTPs), with a biological treatment step (i.e. the activated sludge basin), where a multitude of microorganisms act in concert [1–3]. The activated sludge microbiome consists of thousands of species, a core and transient microbiome consisting of bacteria, archaea, protists, fungi, and even microscopic animals. Due to the large scale and partial openness of the bioreactor to the environment this microbiome underlies fluctuations in community composition and functional efficiency. To cope with and steer this microbiome into a desired direction, WWTP operators commonly manage the microbiome indirectly through a change in physiochemical parameters, like an adjustment of retention time and aeration, or chemical supplementation [4, 5].

Functions fulfilled by the wastewater microbiome are manifold. The coupling of denitrification and nitrification reduces wastewater nitrogen levels, whereas aerobic and anaerobic heterotrophs aid in degrading organic materials [3, 6, 7]. The growth of microbial biomass plus aeration leads to flocculation, facilitating the separation of solids through sedimentation in the following clarification step [2, 8, 9]. One of the most important challenges in the biological step of wastewater treatment, if not the most critical, is sludge bulking, alongside the related issue of sludge foaming (hereafter referred together to as sludge bulking for simplicity). Both disrupt sedimentation and thereby prevent efficient sludge removal [5, 10–12]. As a result, insufficiently treated wastewater may be released into the environment, which is accompanied by a detrimental environmental impact and, in many countries, leading to heavy fining of WWTP operators, if detected [13, 14]. Accordingly, there is a high interest in predicting, treating, and preventing sludge bulking. Common technical control measures are not always successful and most sludge bulking treatments rely on the supplementation of chemical precipitants [10, 11, 14]. Additional precipitant usage as a control measure is expensive, and the use of non-dedicated agents can negatively impact key functional bacteria, including nitrifiers, thereby impairing the functionality of the WWTP [11, 12, 14, 15]. It is thus crucial to better understand microbiome functioning in order to develop more sustainable ways of preventing sludge bulking during wastewater treatment.

Sludge bulking is caused by a bloom of filamentous bacteria, mostly *Proteobacteria, Actinobacteria, Firmicutes, Planctomycetes, Betaproteobacteria*, and *Chloroflexi*. It is recognised when the sludge volume index (SVI) exceeds 150 ml/g [3, 12, 15]. Globally, the most prominent bacterial genus causing sludge bulking events all over the globe is Ca. Microthrix parvicella, belonging to the *Actinobacteria* [11, 16]. To this date, Ca. M. parvicella could not be sufficiently cultured, preventing its formal description and the precise measurement of its physiological capabilities and parameters affecting its abundance [11, 17, 18]. Nonetheless, it is known that Ca. M. parvicella preferentially degrades long-chain fatty acids, grows relatively slowly, and shows its highest abundances in low dissolved oxygen (DO) environments and at colder temperatures (<20°C) [11, 12, 17, 19]. The cause of the negative correlation between its abundance and temperature remains unclear, because it could result either from the physiological constraints of this species’ metabolism or by seasonal ecological interactions.

Predation has been observed to impact the microbial community composition and biomass in WWTPs. Predators affect wastewater prokaryotic diversity, taxon richness, evenness, and beta diversity [2, 20–23]. Heck *et al.* [9] recently showed that the community composition of wastewater predators (i.e. protists and microscopic animals) was tightly linked to changes in temperature. Although wastewater prokaryotes were unaffected by seasonal changes in temperature, their community composition was impacted by the seasonally changing community of temperature-dependent predators. As a result lower variability of the prokaryotic community composition during warm periods was found, most likely due to high predation pressure in wastewater during warmer periods of the year [9, 24].

In case of Ca. M. parvicella, potential biocontrol through predation by rotifers has been intensively investigated. Despite decades of research, rotiferan biocontrol of Ca. M. parvicella has never led to full-scale application, presumably due to culturing problems of rotifers, small effect sizes, and/or their sensitivity to toxins and low temperatures [11, 24–26]. Protistan biocontrol potential of Ca. M. parvicella remains unexplored, as filamentous growth is considered an effective defence mechanism against predation by protists. Recent work by Dumack *et al.* [27] provides evidence that testate amoebae within the Arcellinida (Amoebozoa) have evolved special adaptations for consuming large and filamentous prey. Arcellinida evolved a shell that acts as an anchor for actin of the pseudopodia to pull on and break large and filamentous prey [28–30]. Arcellinida are amongst the most abundant wastewater protists, and although predation on filamentous bacteria was anecdotally reported, their biocontrol potential was never thoroughly assessed [25, 31, 32].

Despite the need to prevent discharge of sludge material into the environment and to reduce the reliance on additional chemical precipitants, no environmentally sustainable biocontrol strategy for sludge bulking is as yet available [10, 11, 14]. This study investigates multiple long-term monitoring datasets from municipal German WWTPs to explore the linkage of *Arcella* spp. and filamentous bacteria in interkingdom wastewater ecology. Special attention was given to filament density, the abundances of filamentous bacteria species, and their correlations with physicochemical and biotic factors. We identified a naturally occurring antagonistic protist to filamentous bacteria and shifted from correlative data exploration to causative, experimental validation and prediction to explore the biocontrol potential of the protist *Arcella* spp.

## Material and methods

### Data collection and preparation

Data were accessed from four WWTPs across Germany, referred to as Datasets 1, 2, 3, and 4 to maintain requested anonymity. Staff trained in morphological identification of wastewater bioindicators gathered the data through regular microscopic examination of samples obtained from aeration tanks, according to the guidelines outlined by the Bavarian Environment Agency [5]. These guidelines recommend the quantification of the abundance of prokaryotic and eukaryotic microorganisms with bioindication capacity as well as the filament density as a rapid assessment of WWTP functionality. The abundance data provided by each of the facilities were reported as categorical values. For testate amoebae, e.g. category 0 represents 0 individuals, category 1 represents 1–5 individuals, category 2 represents 6–10 individuals, and category 3 represents 11 or more individuals per sample. Environmental parameters were considered in our analysis when available, with water temperature being measured consistently across all monitoring datasets. Other parameters varied by dataset: Dataset 1 included pH value, ammonium, chemical oxygen demand, total phosphorus concentration, total bound nitrogen concentration (TNb), total organic carbon (TOC), and filterable substances; Dataset 2 only included pH value, nitrite, nitrate, ammonium, total nitrogen, and TNb; and Datasets 3 and 4 included the SVI. Sampling frequency and duration also differed across datasets, with Dataset 3 being sampled weekly for 35 months, whereas Datasets 1, 2, and 4 were sampled monthly, spanning 102, 66, and 35 months, respectively (Supplementary Table S1).

All statistical analyses were conducted using R version 4.3.1 [33] in RStudio, employing the packages vegan v. 2.6.4 [34], tidyverse v. 2.0.0 [35], dplyr v. 1.1.3 [36], lubridate v. 1.9.3 [37], readxl v. 1.4.3 [38], and zoo v. 1.8.12 [39]. Unless stated otherwise, these packages were used for all data processing and statistical analyses. All scripts and raw data for the subsequently detailed analyses are available on GitHub (Lotka-Volterra-Dynamics-Facilitate-Sustainable-Biocontrol-of-Wastewater-Sludge-Bulking).

### Non-metric multidimensional scaling

Community data were plotted using metaMDS and envfit functions in the vegan package to visualise the strongest drivers of environmental factors and species. Only the top 10 variables with the highest explained variation (*R*^2^ values) and *P* values below .05 were displayed. Given the categorical nature of the measured abundance data, Gower’s distance was selected as the dissimilarity measure. This metric is particularly well-suited for mixed-type data, as it accommodates both continuous and categorical variables, allowing for an effective quantification of differences between samples [40].

### Correlation analysis and heatmap generation

Initial visual inspection of histograms suggested non-normal distributions, which were confirmed through the Shapiro–Wilk test [41]. Due to the non-linear categorical nature of the data, relationships between environmental parameters and microbial abundances were analysed using two different methods (i.e. polychoric and Spearman’s rank correlation) [42]. Both methods show in agreement the same patterns throughout the analyses, which is why only Spearman’s rank correlations are depicted in the plots. Correlations were computed using the cor.test function in R, and the resulting associations were visualised as heatmaps generated with ggplot2 v. 3.4.3 [43] and reshape2 v. 1.4.4 [44].

### Assessment of temporal fluctuations

Lineplots were used to visually assess temporal fluctuations in the long-term Dataset 1 and the replicated dataset. The replicated dataset was obtained by the aggregation of Datasets 1–4 with at least three samples per month covering 2021 to 2023 (*n* = 249), which allowed for a comparison of monthly sampling intervals. Only selected organisms, Ca. M. parvicella, “type 0041-like filamentous bacteria”, *Arcella* spp., rotifers, the “filament density,” and temperature were included in the replicated dataset. In this context, filament density refers to the sum of all individual filaments observed in the sample (German: “Gesamtfädigkeit”) [5], whereas type 0041-like filamentous bacteria represent bacterial filaments of unknown taxonomy, colloquially called “type 0041” and similar morphotypes of unknown taxa [10, 14].

### Structural equation modelling

Structural equation modelling (SEM) was used to test and refine hypothesised models of ecosystem processes and interactions [45]. Models were calculated using the lavaan package v. 0.6.18 [46], and visualised using tidySEM v. 0.2.7 [47].

### Mechanistic background evaluation and laboratory experiment

In addition, direct microscopic investigations to confirm the interaction of *Arcella* spp. with filamentous bacteria were employed. For this, Arcella hemisphaerica was observed in natural samples from (i) a pond (coordinates 52.1253080, 21.0452141) and (ii) the aerated sludge bioreactor of the WWTP in Cologne Weiden, Germany (coordinates 50.9391141, 6.8113971). The latter was enriched with a previously established *A. hemisphaerica* culture derived from the same WWTP to increase cell numbers. Samples were investigated and continuously filmed with a Nikon Eclipse 90i microscope at 600x and differential interference contrast.

A microcosm experiment was conducted to experimentally confirm and quantify the putative antagonistic effect of *Arcella* spp. on the abundance of filamentous bacteria. Wells of a 24-well-plate were inoculated with 1 ml of wastewater and 20 *μ*l of freshly sampled sludge flocs containing filamentous bacteria. The sludge was sampled from the same WWTP in Cologne Weiden, Germany, as described above on 24 June 2025, and processed immediately upon arrival in the lab. Approximately 30 individuals (200 *μ*l) of an A. hemisphaerica culture derived from the same WWTP, were added as predator treatment (*n* > 20) and 200 *μ*l of supernatant culture medium were added to the control (*n* > 20). The microcosms were allowed to sediment for one day before initial documentation (called time point 0 days, i.e. *t_0_*). Changes in optical density (OD) were documented after 6, 14, and 21 days with an inverted microscope and 4x magnification respectively. The pictures were analysed for changes in OD (%) with ImageJ [48]. OD was determined by a subtraction of brown pixels and an area measurement. Differences in OD with respect to *t_0_* were calculated. Multivariate analysis of variance (MANOVA) and t-tests [49, 50] of respective time points were calculated to evaluate changes in OD due to predation.

### Time series analysis

To reduce sampling errors, for the time series analysis, the mean of at least three sampling points from the replicated dataset was calculated to represent each monthly value, resulting in an overall sample size of *n* = 36.

### Time lag analysis

Time lag analysis was employed to disentangle temporal dependencies between *Arcella* spp. and Ca. M. parvicella. The lag2.plot function from the astsa package v. 2.1 [51] was used to visualise the relationship between these variables at different time lags. This analysis calculated correlations between the y-variable (*Arcella* spp*.*) at time t and the x-variable (Ca. M. parvicella) at prior time points, identifying the optimal lag (in months) based on the highest observed correlation values (Fig. S11).

### Vector autoregression

Vector autoregression (VAR) models were constructed from the time series objects to quantify the reciprocal impact of taxa. The R packages tseries v. 0.10.57 [52], vars v. 1.6.1 [53], forecast v. 8.23.0 [54], urca v. 1.3.4 [55], and mFilter v. 0.1.5 [56] were used to perform VAR modelling and data visualisation. Granger causality tests were conducted, using the causality function (package vars) to evaluate whether past densities of Ca. M. parvicella could predict *Arcella* spp. abundance, and *vice versa*. Instantaneous causality was also assessed with this function.

To evaluate species-specific responses to unexpected changes in the other species’ abundance, impulse response function (IRF) analysis was performed using the irf function (package vars) [57]. Additionally, forecast error variance decomposition (FEVD) was employed with the fevd function (package vars), to quantify how much of the variance in prediction errors for one organism could be attributed to changes in the other species’ abundance [58].

## Results

**Figure 1:**
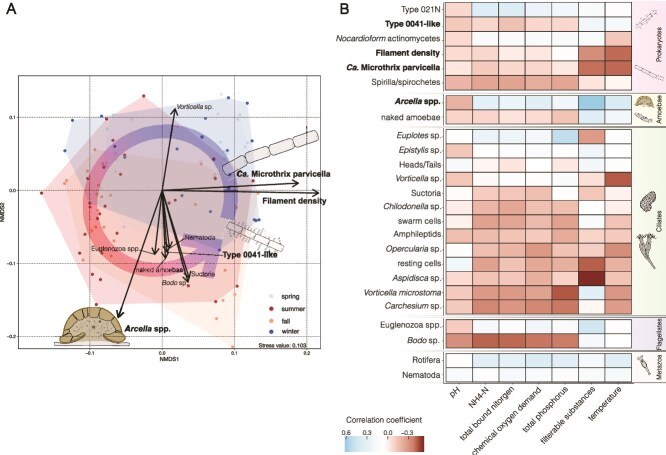
Seasonal dynamics and correlation patterns in the microbial communities of an aerated bioreactor (dataset 1). (A) Non-metric multidimensional scaling (NMDS) plot, with taxonomic organism variables fitted to the ordination space, illustrating the seasonal dynamics of the microbial community, the round arrow illustrates the annual cycle. The colours of the data points indicate the corresponding season (light blue = spring, blue = winter, red = summer, orange = fall). The length of each arrow represents the strength of the association between the corresponding organism and the ordination space. (B) Heatmap depicting the results of Spearman’s rank correlation analysis between the observed taxa and associated environmental metadata. Blue and red indicate positive and negative correlation coefficients, respectively. Note the strong negative correlation between filament density and the abundance of Ca. M. Parvicella and temperature, indicating a temperature dependence of both. Drawings illustrate taxa of particular importance in the subsequent analyses (i.e. Ca. M. Parvicella, type 0041-like filamentous bacteria, and *Arcella* spp.).

Data sampled for almost a decade at a German WWTP revealed that the activated sludge microbiome undergoes regular annual cyclic fluctuations, with a distinct cluster community in the warmer seasons (summer and fall) clearly separated from communities in the colder seasons (winter and spring; Fig. 1A). This pattern indicates a clear change of temperature-dependent recurring annual microbial communities in wastewater. The vector of filament density, a measurement for the abundance of filamentous bacteria, indicated a strong association with colder seasons and the abundance of Ca. M. parvicella, showing a strong dependence of filament density on the abundance of Ca. M. parvicella with season (Figs S1, S2, S5, S8). A subsequent correlation analysis between taxa, the filament density, and environmental variables revealed significant negative correlations with temperature (correlation coefficient (ρ) of about −0.40, respectively), supporting the finding that seasonal temperature fluctuations are the main driver of Ca. M. parvicella abundance and filament density (Figs 1B, S3, S6, S9).

In contrast to most warmer-season-associated taxa, whose vectors pointed in a similar direction, the vector for *Arcella* spp. was distinctly shifted to the opposite direction of the Ca. M. parvicella and filament density vectors. This suggests the presence of an additional strong variable, potentially a biotic relationship, influencing the abundance of *Arcella* spp. positively, even though it negatively affects Ca. M. parvicella and filament density.

**Figure 2:**
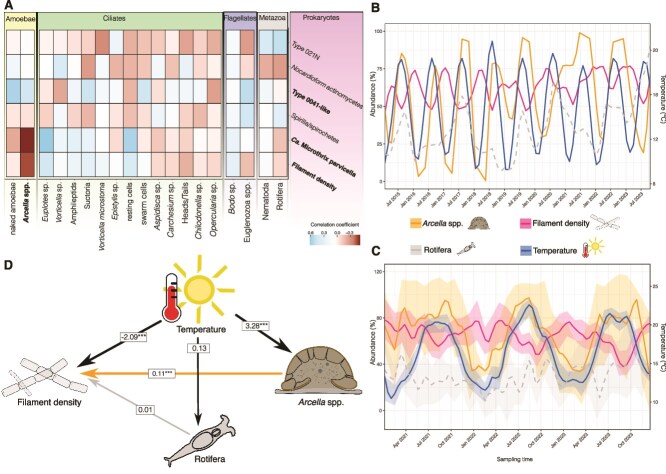
Interactions between *Arcella* spp. and filamentous bacteria in aerated bioreactors. (A) Heatmap depicting the Spearman’s rank correlation analysis results between eukaryotic and prokaryotic taxa from dataset 1. Blue and red indicate positive and negative correlation coefficients, respectively. Note the strong negative correlations between Ca. M. Parvicella, filament density and Arcella spp. suggesting an inverse relationship between the organisms. (B) Lineplot showing the abundance trends of selected organisms from dataset 1. The alternating patterns between Arcella spp., temperature, and filament density suggest contrasting interactions, whereas rotifer abundance does not exhibit a clear trend. (C) Lineplot showing abundance patterns in the replicated dataset where variations are less pronounced, but still visible due to noise and variation. The rotifer abundance remains seemingly random. (D) SEM results demonstrate that temperature acts as the primary driver of changes in filament density and Arcella spp. abundance, whereas Arcella spp. also exerts an independent influence on filament density. Illustrations are provided for Arcella spp., filament density, and rotifers.

In order to investigate whether a direct association between both groups existed, we analysed correlations amongst eukaryotic and prokaryotic taxa (Fig. 2A). Negative correlations between *Arcella* spp. and Ca. M. parvicella, as well as filament density (ρ of −0.24 and −0.20, respectively), were revealed (Figs S4, S7, S10). These negative correlations, even without considering temperature in the analysis, indicate an inverse relationship between *Arcella* spp. and Ca. M. parvicella and filament density.

Regular temperature-dependent fluctuations of filament density and the abundance of *Arcella* spp., occurred (Fig. 2B). *Arcella* spp. abundance, as well as filament density, rise and fall with temperature. Filament density appears to peak during periods when *Arcella* spp. abundances are low. Additionally, rotifers as putative antagonists of filamentous wastewater bacteria, were added. Despite a general positive correlation of rotifer abundance with temperature, the correlation of rotifer abundance with filament density was rather weak.

To provide stronger evidence all datasets were aggregated to obtain a replicated time series of spatially independent WWTPs that exhibit the same annual cycling, although showing more variability (Fig. 2C). SEM was used to disentangle the impact of temperature and the abundance of *Arcella* spp. and rotifers on filament density. The abundance of *Arcella* spp. was positively correlated with increasing temperature (path coefficient (pc) = 3.28) and explains a significant proportion of the overall filament density (pc = 0.11) but temperature affected filament density negatively (pc = −2.09). The positive effect of *Arcella* spp. on filament density may be explained by the nature of time serial data. Note that the path coefficient of 0.11 seems low, but the abundances of both groups are measured as categorical data, meaning that the coefficient must be interpreted as a percentage, e.g. an increase of about 10 abundance categories of *Arcella* spp. corresponds to an increase of one abundance category of the filament density. As no significant effect was found for rotifers, they were excluded from further analysis.

**Figure 3:**
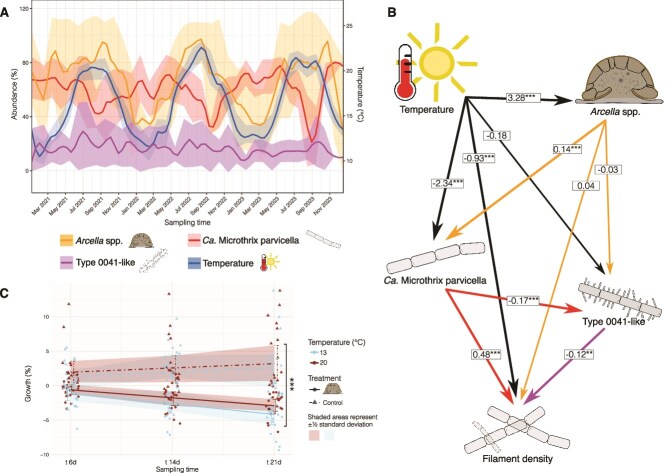
Specific influence of *Arcella* spp. on filamentous bacteria. (A) Lineplot depicting abundance patterns in the replicated dataset, with Ca. M. Parvicella and type 0041-like filamentous bacteria as representatives for filament density. Alternating abundance trends between *Arcella* spp. and Ca. M. Parvicella suggest a potential interaction, whereas type 0041-like filamentous bacteria show no notable interaction here. (B) SEM results for all considered organisms, highlighting temperature as the primary influencing factor on *Arcella* spp. and Ca. M. Parvicella. A significant negative association is observed between type 0041-like filamentous bacteria and filament density, suggesting a limited role in overall filament formation. In contrast, Ca. M. Parvicella shows a significant positive association with filament density, alongside a negative relationship with type 0041-like filamentous bacteria. Additionally, *Arcella* spp. exhibit a positive association with Ca. M. Parvicella abundance, suggesting a specific link between these taxa. (C) Lineplot of the microcosm experiment showing changes in the abundance of filamentous bacteria and floc biomass over time. The *Arcella* spp. treatment samples exhibit significantly reduced filamentous bacterial abundance at the final time point t_21d_, providing strong evidence for *Arcella* spp. mitigating filamentous bacterial growth. Illustrations are provided for *Arcella* spp., Ca. M. Parvicella, filament density, and type 0041-like filamentous bacteria.

The influence of *Arcella* spp. on the filament density was further examined by analysing whether *Arcella* spp. generalistically or selectively affected groups of filamentous bacteria. Only Ca. M. parvicella showed strong seasonal fluctuations (Fig. 3A), whereas the abundance of type 0041-like filamentous bacteria was much more stable, suggesting a closer association of *Arcella* spp. with Ca. M. parvicella. In support, the SEM reveals a significant impact of *Arcella* spp. on Ca. M. parvicella (pc = 0.14), but not on type 0041-like filamentous bacteria. Additionally, type 0041-like filamentous bacteria show a significant negative association with both, filament density (pc = −0.12) and Ca. M. parvicella (pc = −0.17), indicating competitive exclusion and that their contribution to filament density was low. In contrast, Ca. M. parvicella demonstrates a significant positive association with filament density (pc = 0.48). *Arcella* spp. reveals no direct impact on filament density, suggesting that the observed effect (Fig. 2D) is mediated indirectly through the abundance of Ca. M. parvicella. The strong influence of temperature on Ca. M. parvicella (pc = −2.34) further supports that the abundance of Ca. M. parvicella is the main driver of filament density.

**Figure 4:**
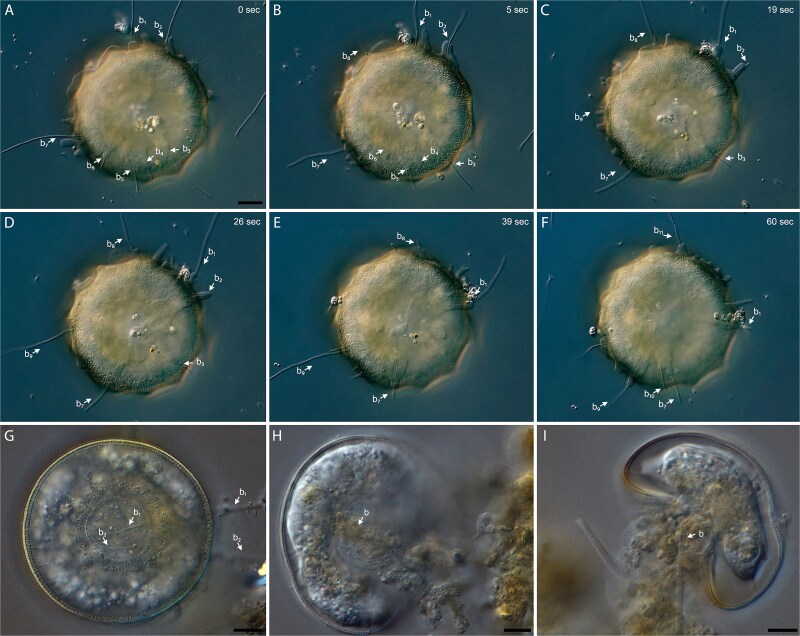
*Arcella hemisphaerica* feeding on multiple filamentous bacteria and sludge flocs. (A–F) a time series spanning over 60 s. Every single bacterial filament is labelled and numbered. (G–I) morphologically different wastewater filamentous bacteria are ingested. A video is provided as Supplementary Video 1.

To estimate the type of interaction, its time delay and impact, a microcosm experiment was set up. During 21 days of incubation at 13°C or 20°C, *A. hemisphaerica* not only suppressed bacterial growth (MANOVA; *P* value <.001), but also reduced the abundance of bacteria and flocs (t-test, *P* value <.001; Fig. 3C). This effect was found irrespective of the environmental temperature. This clearly antagonistic predator–prey relationship between the bacterivorous *Arcella* spp. and the filamentous bacterium Ca. M. parvicella indicates that *Arcella* spp. serves as a natural biocontrol agent mitigating filamentous bacteria. The predator–prey interaction could be confirmed by video documentation A. hemisphaerica consumed up to ten bacterial filaments simultaneously with a handling time of about 30 s each (Fig. 4A–F). A*rcella* hemisphaerica attached to the filament ends with its pseudopodia and subsequently pulled the filaments to the aperture (i.e. the opening of the amoeba’s shell) for phagocytosis. During consumption, some bacterial filaments were bent. The video shows the consumption of one bacterial filament every 3 s by A. hemisphaerica. These numbers represent the maximal observed feeding rate, as feeding in A. hemisphaerica is no uniform process. *In situ* observation of activated sludge samples revealed that A. hemisphaerica ingests several morphologically different bacterial filaments (Fig. 4G–I), including entire attached sludge flocs (Fig. 4H).

**Figure 5:**
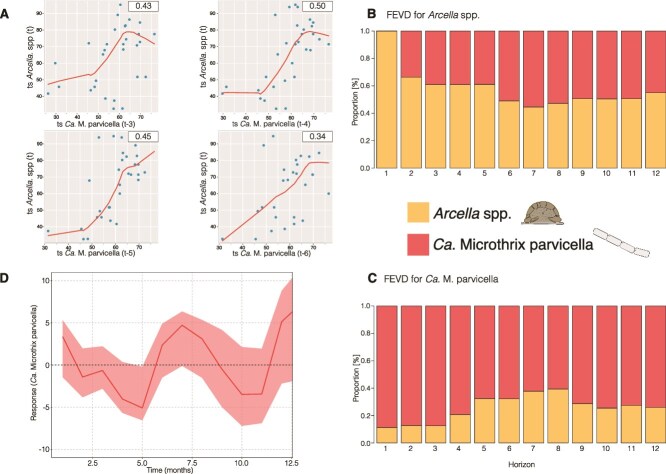
Predator–prey dynamics over time. (A) Lag plots depicting relationships between Ca. M. Parvicella (x-variable) and *Arcella* spp. (y-variable) across different time lags, showing the strongest interactions at time lags t-3, t-4, t-5, and t-6 months. (B, C) FEVD plots for *Arcella* spp. (B) and Ca. M. Parvicella (C), respectively. The variance of prediction errors for both variables is primarily explained by their own previous abundances. The forecast horizon is measured in 1-month intervals along the horizontal axis. (D) IRF showing the response of the Ca. M. Parvicella time series to a shock in *Arcella* spp. abundance. The results indicate a reduction in bacterial abundance over ~5 months. The y-axis quantifies the deviation of the variable from its original equilibrium after the shock.

To disentangle time-dependent correlation and causation, we subjected the data to temporal analyses. Granger causality analysis indicated a directed impact of Ca. M. parvicella on *Arcella* spp. (*P* value = .047), with a marginally supported (*P* value = .09) immediate effect within the same sampling period (i.e. within the same month). Accordingly, we tested for a delayed response to further define the type of association between *Arcella* spp. and Ca. M. parvicella. The time lag analysis identified a long-time (annual), temporal relationship between Ca. M. parvicella and *Arcella* spp. abundances, showing a positive correlation between the abundances of Ca. M. parvicella and *Arcella* spp. at negative time shifts of 3–6 months (i.e. _t-3_, _t-4_, _t-5_, and _t-6_; Fig. 5A, Fig. S11). These findings suggest that strong changes in Ca. M. parvicella abundance precede changes in *Arcella* spp. abundance by ~3–6 months and an immediate impact within 1 month is weakly supported.

We investigated how changes in the abundance in one of the populations affect the other over time. FEVD analysis revealed that up to 55.4% of the variance in *Arcella* spp. was explained by the prior abundance of Ca. M. parvicella, highlighting a substantial influence of Ca. M. parvicella on *Arcella* spp. (Fig. 5B). Conversely, up to 39.4% of the variance in Ca. *M. parvicella* was attributed to the prior abundance of *Arcella* spp., indicating a notable but somewhat lower explanatory power of *Arcella* spp. on Ca. M. parvicella.

It seems that the abundance of Ca. M. parvicella has a strong influence on the abundance of *Arcella* spp., which in turn affect Ca. M. parvicella to a measurable, but weaker extent. To estimate the biocontrol potential, we subjected the data to an IRF analysis (Fig. 5C). The model supports a biocontrol potential against an overgrowth of Ca. M. parvicella by an addition or increase of *Arcella* spp. abundance through a predicted immediate decline in the abundance of Ca. M. parvicella lasting for ~5 months.

## Discussion

### The lynx and the hare

In the early 20th century, biologist Charles Gordon Hewitt observed oscillating, multi-year lagged patterns in the abundances of predatory Canadian lynx and their prey, snowshoe hares, using data derived from fur sales records of the Hudson’s Bay Company. These dynamics followed the dynamics predicted by the Lotka–Volterra equation [59, 60], and have since become a fundamental example illustrating the principles of predator–prey interactions in ecology. This foundational work has led to the development of more complex models and numerous applications in ecology-based management processes, such as integrated pest management [57, 61, 62]. Based on this background, we were able to leverage large-scale data spanning over multiple years and statistical tools developed for time series ecology to showcase the biocontrol potential of *Arcella* spp. against Ca. M. parvicella and sludge bulking. The lynx and hare, whose populations are interconnected demonstrate how predation pressure and resource availability interact to shape population cycles [63, 64]. Similarly, our findings reveal that Ca. M. parvicella and *Arcella* spp. exhibit a comparable predator–prey dynamics, mediated by seasonal changes in temperature [9, 65, 66].

Currently, effective strategies to control Ca. M. parvicella sludge bulking in WWTPs are limited. Existing approaches, such as reducing sludge retention time (SRT) and controlling DO concentrations [16, 18], as well as the use of chlorine, polyaluminium chloride products (PAX16 and PAX18), nickel as an heavy metal inhibitor, and synthetic polymers, most come with significant drawbacks [67–70]. These include unintended impacts on beneficial bacteria, short-lasting effects, and serious environmental side effects [11–13, 71, 72]. Ozone treatment and magnetic field applications have also been explored, but require further evaluation [12, 73]. Altogether, these limitations highlight the benefit of an efficient, biological, long-term, and cost-effective solution for controlling Ca. M. parvicella.

Our findings suggest that promoting natural predators of Ca. M. parvicella could offer an environmentally friendly alternative for managing microbial communities in WWTPs. Ca. M. parvicella reveals its highest abundances at colder temperatures, due to reduced predation pressure by *Arcella* spp. that are sensitive to low temperature [11, 12, 17, 19]. Time lag analysis suggested that Ca. M. parvicella and *Arcella* spp. populations follow the annual cycle tightly as they are lagged by 3–6 months. The time lag analyses as well as the positive path coefficients in our models demonstrate that changes in the abundance of *Arcella* spp. follow the changes of abundance of Ca. M. parvicella under natural conditions. Accordingly, simple increase of SRT to increase *Arcella* spp. abundances is likely to be accompanied by further growth of Ca. M. parvicella [7, 18, 74]. Instead of a simple increase of SRT, we show that the abundance of *Arcella* spp. must be increased for effective treatment, for instance by the addition of cultured material. An immediate effect of Ca. M. parvicella abundances within the same sampling month is supported only weakly with a *P* value of less than 0.1. Despite this weak statistical support of an immediate effect within a single month based on environmental data, our experiment confirms that after 6, 14, and 21 days subsequent to supplementation, A. hemisphaerica did not only lead to a suppression of filament and floc growth, but a significant decline. IRF analyses indicates that after biocontrol application of *Arcella* spp., a long-lasting effect, up to 5 months, could be achieved. Collectively, these findings suggest an effective biocontrol based on a naturally occurring Lotka–Volterra predator–prey dynamic in the aeration tanks of WWTPs by supplementation.

Our findings extend previous work on rotifers as biocontrol agents [26, 75]. In contrast to rotifers, *Arcella* spp. demonstrate consistent, long-term associations with Ca. M. parvicella populations across full-scale WWTPs, suggesting greater potential for practical application. Now, the next step to this research is a proof of concept of biocontrol on a full-scale WWTP with industrial-scale addition of *Arcella* spp. or engineering facilities to specifically increase naturally occurring *Arcella* spp. abundances. During these investigations, we recommend examining potential side effects caused by a high grazing pressure of *Arcella* spp. on sludge composition, abundance and density of flocs, subsequent affection of sedimentation behaviour, and subsequent effects on other predators or pathogens, such as specific viruses for each group.

### Concluding remarks

Promoting *Arcella* spp. populations in treatment systems could provide a sustainable solution to manage sludge bulking and improve treatment efficiency without harmful chemical additives. Future research should focus on full-scale trials to validate *Arcella* spp.-based biocontrol in WWTPs and explore optimal conditions for *Arcella* spp. proliferation. By integrating these biological control strategies, WWTPs could reduce sludge bulking, leading to more resilient and efficient wastewater treatment processes and cost efficiency.

## Supplementary Material

Supplementary_Material_acceptance_wraf235

Supplementary_Video_1_Arcella_feeding_4x_speed_wraf235

## Data Availability

The raw data and used code are available in the Lotka–Volterra Dynamics Facilitate Sustainable Biocontrol of Wastewater Sludge Bulking repository on GitHub [https://github.com/fabib1209/Lotka-Volterra-Dynamics-Facilitate-Sustainable-Biocontrol-of-Wastewater-Sludge-Bulking/tree/main].
